# The Epidemiology and Health Burdens of Influenza Infections Amongst Hospitalized Children Under 5 Years of Age in Jordan: A National Multi-Center Cross-Sectional Study

**DOI:** 10.3390/vaccines13010012

**Published:** 2024-12-26

**Authors:** Munir Abu-Helalah, Samah F. Al-Shatnawi, Mohammad Abu Lubad, Enas Al-Zayadneh, Mohammad Al-Hanaktah, Mea’ad Harahsheh, Montaha AL-Iede, Omar Nafi, Ruba Yousef, Ihsan Almaaitah, Mai Ababneh, Toqa AlZubi, Rand Abu Mahfouz, Heba Adaylah, Hamzeh AlHajaj, Mohammad Al Tamimi, Simon B. Drysdale

**Affiliations:** 1 Department of Family and Community Medicine, Faculty of Medicine, University of Jordan, Amman 11942, Jordan; 2 Public Health Institute, The University of Jordan, Amman 11942, Jordan; 3 Department of Clinical Pharmacy, Faculty of Pharmacy, Jordan University of Science and Technology, P.O. Box 3030, Irbid 22110, Jordan; sfshatnawi@just.edu.jo; 4 Department of Microbiology and Pathology, Faculty of Medicine, Mutah University, Karak 61710, Jordan; abu_lubbad@mutah.edu.jo; 5 Department of Pediatrics, Faculty of Medicine, University of Jordan, Amman 11942, Jordan; e.alzayadneh@ju.edu.jo (E.A.-Z.); m.al-iede@ju.edu.jo (M.A.-I.); 6 Faculty of Medicine, University of Jordan, Amman 11942, Jordan; malhanaktah49@gmail.com; 7 School of Pharmacy, University of Jordan, Amman 11942, Jordan; mya9220222@ju.edu.jo; 8 Department of Pediatrics, Faculty of Medicine, Mutah University, Karak 61710, Jordan; omarnafi@mutah.edu.jo; 9 Medical Department, MENA Center for Research and Development, Amman 11941, Jordan; ruba.yousef1995@gmail.com; 10 Pediatrics Department, Zarqa Governmental Hospital, Zarqa 13112, Jordan; ihsanalmaaitah66@gmail.com (I.A.); mdhamzehhjj@gmail.com (H.A.); 11 MENA Center for Research and Development, Amman 11942, Jordan; mai.ababneh97@gmail.com (M.A.); toqaalzoubi66@gmail.com (T.A.); randabumahfouz@gmail.com (R.A.M.); hebaadaileh56@gmail.com (H.A.); 12 Independent Researcher, Amman 11941, Jordan; matmat1978@yahoo.com; 13 Oxford Vaccine Group, Department of Pediatrics, University of Oxford, Oxford OX1 2JD, UK; simon.drysdale@paediatrics.ox.ac.uk; 14 The NIHR Oxford Biomedical Research Centre, Oxford OX3 7JX, UK

**Keywords:** influenza, clinical, epidemiology, children, below age of five, Jordan

## Abstract

Background/Objectives: Seasonal influenza is a significant global health concern, causing substantial morbidity and mortality, particularly among high-risk groups such as children under five years old. There is scarce local evidence from developing countries such as Jordan on the burden of influenza, which has limited preventive measures. This multi-center national cross-sectional study aimed to assess the epidemiological and clinical burden of influenza among hospitalized children under five years old in Jordan. Methods: Data were collected from 1000 participants across four hospitals between November 2022 and April 2023. Nasopharyngeal specimens were analyzed using multiplex RT-PCR to determine positivity for influenza A and B. Results: We found a 9.9% positivity rate, predominantly influenza A (8.4%), while influenza B was positive among 1.5% of the participants. Positivity rates were higher in older age groups, particularly children older than 2 years. Influenza-positive cases exhibited longer fever durations and higher rates of sore throat. There were no positive influenza cases among participants if they or any of their family members received the influenza vaccine, highlighting the vaccine’s protective role. Logistic regression analysis identified maternal smoking during pregnancy as a significant predictor of influenza positivity. Conclusions: The findings of this study underscore the need for enhanced vaccination efforts and public health policies targeting young children and pregnant women in Jordan. Expanding vaccination uptake could significantly mitigate the burden of influenza and its complications in this vulnerable population.

## 1. Introduction

Seasonal influenza is an acute respiratory infection caused by influenza viruses, which are highly contagious and circulate worldwide. It gives rise to an estimated 3 to 5 million cases of severe illness and about 250,000 to 500,000 deaths globally each year [1].

It has been established that influenza significantly impacts outpatient visits and hospitalizations for children under five years old. According to one study, this age group experiences considerable healthcare use due to influenza [2]. The geographical setting also plays a role, with hospitalization rates for influenza being three times lower in industrialized nations compared with developing countries [3]. Additionally, a systematic review and meta-analysis from China revealed that children under five years old face the highest rates of influenza-related hospitalizations, markedly increasing the overall morbidity and mortality associated with the virus [4].

Vaccination against influenza is one of the key steps to reduce the substantial health and economic burden that seasonal influenza imposes. Even though the influenza vaccine is readily available, vaccination uptake rates are often low, particularly in developing countries [1]. Influenza tends to cause epidemics with serious illnesses and death among high-risk groups such as children less than five years old. According to the US Centers for Disease Prevention and Control (CDC) guidance, everyone six months old and above is recommended to receive the seasonal influenza vaccine annually [5].

Influenza vaccine uptake among children younger than the age of 18 in Jordan, who are 3.8 million in number, contributing to 40% of the population, [6] has low rates of around 10.9%. This is an expected figure because the influenza vaccine is not included as part of the routine immunization schedule in Jordan, either for all children or for high-risk children aged 6 months to five years. Moreover, the influenza vaccine is not given routinely in Jordan during pregnancy to protect children antenatally or during the first 6 months postnatally [7].

In order to prevent influenza burden among children younger than age of 5 years, it is essential to obtain updated epidemiological and clinical data on the burden of influenza among hospitalized children younger than five years old.

## 2. Materials and Methods

### 2.1. Study Design

This multi-center cross-sectional study design involved four study centers from the central, northern, and southern regions of Jordan as follows: (1) Princess Rahma Hospital for Children, Irbid; (2) Zarqa Hospital, which serves the center and east of Jordan; (3) Jordan University Hospital, Amman; and (4) Al Karak Public Hospital, Al Karak.

The primary outcome of this study was to measure influenza positivity among hospitalized children under 5 years old with respiratory symptoms. The secondary outcomes were the clinical characteristics of influenza infections, namely, the symptoms, signs, and complication rates, duration of hospitalization, need for intensive care unit (ICU) admission, and risk factors for hospitalization due to influenza. These outcomes were compared between influenza-positive and influenza-negative cases and between patients with influenza-A and influenza-B infection.

During the time period between 15 November 2022 and 14 April 2023, eligible patients presented with respiratory symptoms in the inpatient wards at the four study sites, according to the criteria below.

The recruitment of patients took place during weekdays and continued during weekends and holidays to support diversity in the access to enrollment.

### 2.2. Inclusion Criteria

The inclusion criteria included patients that permanently resided in the study areas and were hospitalized with an acute respiratory infection according to the below definition and criteria.

### 2.3. Case Definition [8]

Diagnosis of acute respiratory infection was defined as “an illness presenting with one or more of the following symptoms for less than 7 days: Fever (≥38 °C) or hypothermia (<35.5 °C), cough, earache, nasal congestion, rhinorrhea, sore throat, vomiting after coughing, crackles, and labored, rapid or shallow breathing”.

### 2.4. Study Population

Children < 5 years old admitted to the study sites with the following:

(1) At least one sign of an acute infection (temperature ≥ 38 °C or <35.5 °C, abnormal white blood cell [WBC] count or abnormal differential).
(2) Diagnosis of acute respiratory infection as defined above.

### 2.5. Exclusion Criteria

Not a permanent resident of Jordan.

### 2.6. Sample Collection and Processing

Nasopharyngeal (NP) specimens were collected from each patient who met the inclusion criteria and consented to this study.

### 2.7. Microbiology

#### Sample Collection and Transport

Flocked swabs with plastic shafts were used to collect nasopharyngeal specimens from each patient who met the inclusion criteria. The swabs were then inserted into sterile viral transport media (VTM) and immediately placed on refrigerant gel packs or held at 4 °C prior to transport to the laboratory on the day of collection. Upon arrival at the laboratory, the specimens were immediately processed. Viral nucleic acid was extracted from the specimens and stored at −80 °C for further analysis to identify the target viruses.

Multiplex RT-PCR (TaqPath™ Combo Kit-Thermofisher, Waltham, MA, USA) was performed on each nasopharyngeal specimen by testing for respiratory syncytial virus and influenza using the QuantStudio 5 instrument (Applied Biosystem, Foster City, CA, USA) according to the manufacturer’s instructions. Influenza types A and B were then detected using a VIASURE Real-Time Detection Kit (CerTest Biotec S.L., Zaragoza, Spain). The influenza type RT-PCR cycles were as follows: 1 cycle for reverse transcription for 15 min at 45 °C, initial denaturation for 2 min at 95 °C, 45 cycles of denaturation for 10 s at 95 °C, and 45 cycles of annealing/extension for 50 s at 60 °C [9,10].

### 2.8. Power/Sample Size

We expected to enroll 1000 subjects who matched the above clinical criteria. According to previous studies, 6.2% to 18.8% of hospitalized children meeting our eligibility criteria were positive for influenza. [11] Therefore, we expected 10–15% of the subjects to be positive for influenza when calculating the sample size. One thousand participants were needed to obtain a power of 90% with margin of error 5%.

### 2.9. Statistical Methods

Statistical Package for the Social Sciences (SPSS) version 23 was used to analyze the data. Descriptive statistics (Student’s *t*-test and chi-squared test) were used to analyze and compare the categorical variables: demographic characteristics, including the patient characteristics; risk factors; and vaccination details. A binary logistic regression analysis using backwards stepwise elimination was performed to identify predictors of influenza positivity and predictors of complications.

### 2.10. Case Report Form (CRF)

Each study participant had a unique case ID throughout the study. The first part of the form included the inclusion criteria for this study, as described above. The parents/guardians of eligible patients were asked whether they consented to take part in this study after explaining the study details. All were reassured that participation was voluntary and would not affect the service provided. There was a daily site report that counted the number of admissions; eligible patients; and included and excluded patients, including the reasons for not participating.

### 2.11. The Case Report Form (CRF) Fields

#### 2.11.1. Background, Demographic, and Societal Data for Patients and Parents

Sex, age, parents’ ages and educational statuses, special diet/milk, number of people in the household with age groups, patients or siblings attending a kindergarten, and parents or siblings with a history of asthma or eczema. Furthermore, a detailed smoking history was obtained, including the mother’s history of smoking during pregnancy; the father’s, mother’s, or other household members’ smoking (inside or outside the home); and the number of cigarettes per day or waterpipe per week.

#### 2.11.2. Medical History, Including Birth History, Existing Medical Conditions, and Current Regular Medications

Weight, height, birth weight, gestational age at birth, method of delivery, meconium-stained liquor, neonatal intensive care unit (NICU) admission and ventilation status, surfactant, breast feeding, presence of asthma, cystic fibrosis, bronchopulmonary dysplasia, neuromuscular disease, congenital heart disease, other congenital disease, immunodeficiency, eczema (atopy), and other comorbidities were included. Any previous hospital admission with the diagnosis, duration, and dates were included. Vaccine status for the pneumococcal conjugate vaccine (PCV), due to be included in the national immunization program from January 2025, influenza, and SARS-CoV-2 for the patient, mother, father, and siblings were obtained. Regular medications and their indications were also included.

#### 2.11.3. Presenting Symptoms and Signs

Symptoms and their duration prior to admission were included. Other clinical manifestations, such as cardiovascular manifestations, dehydration, wheeze, cyanosis, low activity level, hypoxia (SpO_2_ < 92%), tachypnea, pneumothorax/atelectasis, apnea > 10 s, subcostal/intercostal retractions, and nasal flaring.

#### 2.11.4. Laboratory Findings

Laboratory findings, including white blood cell count (WBC) and differential, blood gas, respiratory viral PCR results, chest X-ray findings on arrival, and bacterial or fungal coinfection based on clinical presentation and positive blood culture results.

#### 2.11.5. Healthcare Prior to Hospitalization at Public or Private Hospitals and Clinics

This also covered care provided at the emergency department at the same hospital prior to admission.

All utilized medicines during the admission, including the name, route, dose, frequency, and number of days during admission, were recorded for every patient. This included all discharge medications, if prescribed.

## 3. Results

### 3.1. Inpatient Data

There were 3580 admissions at study sites during the recruitment phase between 15 November 2022 and 14 April 2023. In total, 1755 children were screened, and 1008 were eligible for the study; eight of them were not included due to failure of the parents to sign the consent form. Thus, 1000 individuals were enrolled in this study.

The median age of study participants was 9.68 (Interquartile range: 3.13–29.83) months, while the mean age was 17.10 (SD: 16.57) months. 59% of participants were male and 68% of them were younger than 2 years.

**Figure 1:**
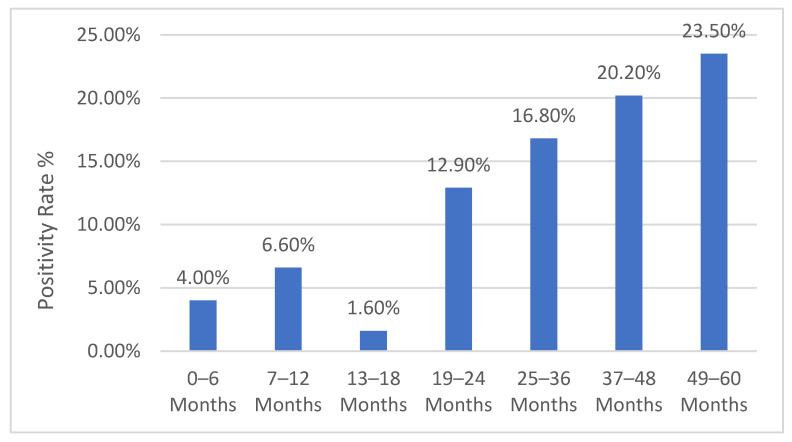
Influenza positivity rate by age group.

Ninety-nine (9.9%) participants tested positive for influenza. The highest positivity rate was reported in children 49 to 60 months old, followed by children 37 to 48 months old. The positivity rate was less than 7% for children younger than 18 months old (Figure 1). Positivity rates by month of recruitment are shown in Supplementary Table S1. The highest positivity rates were reported in December while the lowest were in January.

Further analysis of influenza subtypes revealed that influenza A was dominant with 84 (84%) of 99 influenza-positive cases, leading to a positivity rate of 8.4% for influenza A and 1.5% for influenza B.

There was a statistically significantly lower positivity rate for younger age groups when comparing children ≤ 6 months old with older children (4.0% vs. 13.4%, *p* < 0.001) or children ≤ 2 years old with those older than 2 years (5.6% vs. 19.2%, *p* < 0.001). Supplementary Table S2 shows influenza positivity rates by socioeconomics and medical history. There was no statistically significant difference between influenza-positive and influenza-negative cases in the frequency of studies risk factor.

Table 1 shows a very low uptake of the influenza vaccine amongst study participants and their families. Interestingly, there were no influenza cases if patients or their family members received the influenza vaccine. There were seven (0.7%) participants, 17 fathers, nine mothers, and nine siblings who reported receiving the influenza vaccine.

In the analysis of clinical symptoms (Table 2), it was evident that individuals who tested positive for influenza had a significantly greater prevalence of sore throat when compared with those who tested negative. Conversely, there were statistically lower percentages of individuals reporting breathlessness, wheezing, and tachypnea among the influenza-positive cases. Regarding duration of symptoms (Table 3), it was noted that individuals who tested positive for influenza had a significantly longer mean duration of fever compared with those who tested negative (5.2 days versus 3.8 days, *p* < 0.001).

Supplementary Table S3 shows the clinical manifestations and complications by influenza positivity. There were no statistically significant differences between influenza-positive and influenza-negative cases except for higher rates of tachypnoea and the need for non-invasive ventilation for influenza-negative cases.

### 3.2. Predictors of Influenza Positivity

Table 4 shows results of the logistic regression analysis for predictors of positivity for influenza. Mother’s reported history of smoking during pregnancy was the strongest statistically significant variable followed by presence of leukocytosis. Regarding clinical features, presence of sore throat and longer duration of fever or sore throat were negatively associated with influenza positivity.

### 3.3. Predictors of Complications

Socio-demographic factors, medical history, and clinical features were included in the binary logistic regression analysis for predictors of the presence of at least one complication, need for ICU admission, or duration of stay. None of the included variables were of statistical significance for influenza-positive cases.

## 4. Discussion

Our study revealed that the seasonal influenza virus is an important pathogen leading to a significant burden of hospitalization amongst children in Jordan. The positivity rate of 9.9% is consistent with data from previous studies of developing and developed countries [3,12,13].

The presence of chest infiltrates amongst two thirds of influenza-positive cases goes with the reported data on influenza positivity amongst children with acute lower respiratory tract infections (LRTIs). These figures highlight the local and global burden of influenza amongst this age group, who have been considered at high risk of influenza complications [14]. Global data from 2018 show that there are approximately 20 million episodes of influenza virus-associated LRTI annually along with an estimated 28,000 to 111,000 deaths in children under 5 years old. A systematic review of the global burden of influenza in 2018 revealed influenza accounted for 7% of ALRI cases, 5% of ALRI hospitalizations, and 4% of ALRI deaths in children younger than five years old [11].

An interesting finding in our study is that no participants were positive for influenza if the participants or a family member received the influenza vaccine. This emphasizes the public health impact of vaccination in the prevention of influenza in developing countries such as Jordan. Despite the huge burden of influenza in younger children worldwide, several reports have revealed that only 10–13% of low-income countries (LICs) and lower-middle-income countries (LMICs) have implemented national policies for maternal or child influenza immunization [15,16,17]. A recent study from Jordan revealed that only 2.1% of women had received influenza vaccination during their most recent pregnancy [18]. Another study from Jordan showed that 10.9% of participants had vaccinated their children during the previous season (2015/2016) [7]. These figures are lower than those reported from other regional countries such as Saudi Arabia [19]. The influenza vaccine is not offered freely for children aged 6 months to 5 years or their parents in Jordan, except for patients insured by the Ministry of Health, if they have chronic cardiac or respiratory disease or if they have primary or second immune deficiency [20].

In the analysis of clinical symptoms, fever, cough, and rhinorrhea were the most reported symptoms amongst influenza-positive cases, while sore throat, breathlessness, wheezing, and tachypnea were not associated with positivity of influenza. These figures are consistent with recent data from the USA, where high fever, cough, and rhinorrhea were the most frequently recorded symptoms [20].

Further analysis of influenza subtypes revealed that influenza type A predominated amongst study participants. These data are consistent with findings from previous studies. This is consistent with the global distribution of influenza cases [21,22]. Our data revealed that there were no statistically significant differences in the clinical manifestations between influenza A- and influenza B-positive patients. Our results are consistent with recent figures indicating that the clinical course of influenza B is largely similar to influenza A, typically including fever, cough, and headache [23]. Moreover, a systematic review of the severity and clinical features of influenza showed that viral type or subtype is not usually a predictor of the clinical presentation or disease severity overall [23].

Smoking during pregnancy was the most important risk factor for positivity for influenza amongst our study population. A cross-sectional study from the North of Jordan included 436 pregnant women. It showed that 2.9% of pregnant women quit smoking once pregnancy was confirmed, while 17.6% continued to smoke [24]. Tobacco smoking during pregnancy is well established as a risk factor for reduced immunity and increased risk of infections in infants [25,26]. In countries such as Jordan with high tobacco smoking rates, much work is needed to control smoking during pregnancy.

Most other risk factors for respiratory illness were not statistically significant between influenza-positive and -negative patients. This is consistent with previous findings from Europe, where across all age groups, most children hospitalized for influenza were previously healthy [27,28]. This emphasizes the importance of providing the influenza vaccine for all children 6 months to five years old and during pregnancy to reduce the risk of influenza infections amongst these cohorts [14].

This study included a large sample of 1000 participants from representative sites in Jordan and showed almost 10% of participants were influenza-positive. However, we had several limitations. This study was conducted during the influenza season in Jordan between November 2022 and April 2023. Due to budget constraints, we could not recruit during the whole year, so we may have missed some cases outside the main season. We did not ask about details related to uptake of the influenza vaccine such as barriers and knowledge. This is being conducted through another ongoing national study.

## 5. Conclusions

This national multi-center cross-sectional study in Jordan offers a robust exploration of the epidemiology and health impacts of influenza infections among children under 5 years of age and revealed a substantial burden of influenza. Due to the high positivity rate of 9.9% amongst hospitalized children and associated complications, it is essential that different stakeholders in Jordan work on routine influenza vaccination for children 6 months to five years old and for women during pregnancy. It is also recommended that future public health programs in Jordan focus on improving the uptake of influenza vaccines amongst these groups.

## Figures and Tables

**Table 1:** Investigating “influenza vaccine status” and positivity for influenza.

			Influenza Result		Total	*p* Value *
			Negative	Positive		
Mother received influenza vaccine	NO	Count	892	99	991	0.318
		% within Influenza	99.0%	100.0%	99.1%	
	YES	Count	9	0	9	
		% within Influenza	1.0%	0.0%	0.9%	
Father received influenza vaccine	NO	Count	884	99	983	0.168
		% within Influenza	98.1%	100.0%	98.3%	
	YES	Count	17	0	17	
		% within Influenza	1.9%	0.0%	1.7%	
Patient received influenza vaccine	NO	Count	894	99	993	0.379
		% within Influenza	99.2%	100.0%	99.3%	
	YES	Count	7	0	7	
		% within Influenza	0.8%	0.0%	0.7%	
Siblings received influenza vaccine	NO	Count	892	99	991	0.318
		% within Influenza	99.0%	100.0%	99.1%	
	YES	Count	9	0	9	
		% within Influenza	1.0%	0.0%	0.9%	
		% within Influenza	90.8%	92.9%	91.0%	

* Statistically significant at *p* < 0.05.

**Table 2:** Presence of clinical symptoms by influenza positivity.

	Influenza Result				*p* Value *
	Negative		Positive		
Symptoms:	Count	%	Count	%	
Fever	889	98.7%	99	100.0%	0.248
Cough	866	96.1%	96	97.0%	0.673
Sore throat	154	17.1%	30	30.3%	0.001
Rhinorrhea	506	56.2%	61	61.6%	0.298
Nasal congestion	408	45.3%	46	46.5%	0.823
Poor Feeding	442	49.1%	51	51.5%	0.642
Hypoxia/Cyanosis	253	28.1%	27	27.3%	0.865
Breathlessness	396	44.0%	32	32.3%	0.026
Respiratory crackles	519	57.6%	47	47.5%	0.054
Apnea > 10 s	55	6.1%	7	7.1%	0.705
Wheezing	403	44.7%	33	33.3%	0.030
Low activity level	480	53.3%	57	57.6%	0.415
Tachypnea	320	35.5%	22	22.2%	0.008
Post Tussive Vomiting	301	33.4%	40	40.4%	0.163

* Statistically significant at *p* < 0.05.

**Table 3:** Duration of symptoms by influenza positivity.

Symptoms	Influenza Result				*p* Value *
	Negative		Positive		
	Mean	Standard Deviation	Mean	Standard Deviation	
Fever (days)	3.82	3.640	5.22	4.308	<0.001
Cough (days)	5.43	5.676	5.69	4.991	0.664
Sore throat (days)	0.94	2.839	1.40	2.587	0.118
Rhinorrhea (days)	3.05	4.406	3.14	3.982	0.836
Nasal congestion (days)	2.57	4.587	2.47	4.183	0.846
Poor Feeding (days)	1.73	2.744	2.07	2.822	0.241
Hypoxia/Cyanosis (days)	0.99	3.168	1.32	3.562	0.329
Breathlessness (days)	1.67	2.746	1.38	3.043	0.336
Respiratory crackles (days)	2.37	3.276	2.26	3.439	0.764
Apnea > 10 s (days)	0.21	1.460	0.34	2.191	0.415
Wheezing (days)	1.67	2.623	1.57	3.160	0.724
Low activity level (days)	1.92	2.786	2.17	2.847	0.399
Tachypnea (days)	1.18	2.177	0.77	2.004	0.075
Post Tussive Vomiting (days)	1.00	1.972	1.51	2.655	0.021
Other symptoms (days)	0.55	1.891	0.88	1.880	0.105

* Statistically significant at *p* < 0.05.

**Table 4:** Binary logistic regression analysis of factors associated with influenza positivity (across all age groups).

	B	*p* Value	Odds Ratio
Fever duration (days)	−0.111	<0.001	0.895
Sore throat (Yes/No)	−0.952	0.030	0.386
Sore throat duration (days)	0.174	0.020	1.190
Mother’s reported history of smoking during pregnancy			
YES	2.152	0.039	8.604
NO	reference		
White blood cell count (×10^9^/L)			
<4	−0.761	0.332	0.467
>10	1.508	<0.001	4.518
4 to 10	reference		

## Data Availability

The original contributions presented in this study are included in the article/supplementary material; further inquiries can be directed to the corresponding author.

## Supplementary Materials

The following supporting information can be downloaded from https://www.mdpi.com/article/10.3390/vaccines13010012/s1. Table S1. Influenza positivity by month; Table S2. Demographic factors associated with influenza results; Table S3. Descriptive statistics of clinical findings by influenza positivity.
