# Supplementary material for: The Epidemiology and Health Burdens of Influenza Infections Amongst Hospitalized Children Under 5 Years of Age in Jordan: A National Multi-Center Cross-Sectional Study
Source: Vaccines (Basel). 2024 Dec 26;13(1):12. doi: 10.3390/vaccines13010012 (PMC11769506; doi:10.3390/vaccines13010012)
Supplement: Supplementary file 1 [file vaccines-13-00012-s001.zip › vaccines-3367941-supplementary.pdf]

Supplementary Table s1. Influenza positivity by month

			Influenza		Total	P-value*
			NO	YES		
**Date month**	Nov-2022	Count	329	33	362	**0.016**
		% within Month	90.88%	9.12%	100%	
	Dec-2022	Count	534	63	597	
		% within Month	89.45%	10.55%	100%	
	Jan-2023	Count	222	8	230	
		% within Month	96.52%	3.48%	100%	
	Feb-2023	Count	71	5	76	
		% within Month	93.42%	6.58%	100%	
	Mar-2023	Count	78	4	82	
		% within Month	95.12%	4.88%	100%	

***Chi-square, statistically significant at P < 0.05.**

Supplementary Table S2. Demographic factors associated with influenza results.

		Influenza results				
		Negative (n= 901)		Positive (n= 99)		
		Count	Row Valid N %	Count	Row Valid N %	P-value*
**Gender**	Female	378	91.30%	36	8.70%	0.284
	Male	523	89.25%	63	10.75%	
**Region**	Middle	440	88.00%	60	12.00%	0.064
	North	233	93.20%	17	6.80%	
	South	228	91.20%	22	8.80%	
	Total	901	90.10%	99	9.90%	
**City**	Amman	223	89.20%	27	10.80%	0.097
	Irbid	233	93.20%	17	6.80%	
	Karak	228	91.20%	22	8.80%	
	Zarqa	217	86.80%	33	13.20%	
	Total	901	90.10%	99	9.90%	
**Living area**	Rural	300	91.74%	27	8.26%	0.225
	Urban	601	89.30%	72	10.70%	
	Total	901	90.10%	99	9.90%	
**Preterm or Full term**	full term	764	90.63%	79	9.37%	0.195
	preterm	137	87.26%	20	12.74%	

**WGA (Gestational Age in Weeks)**	<29	1	50.00%	1	50.00%	0.119
	29-30	3	75.00%	1	25.00%	
	31-32	15	93.75%	1	6.25%	
	33-34	24	96.00%	1	4.00%	
	35-36	94	85.45%	16	14.55%	
	>=37	764	90.63%	79	9.37%	
**Delivery**	Caesarean section	416	89.27%	50	10.73%	0.711
	Normal VD	485	90.82%	49	9.18%	
**Meconium-stained liquor**	NO	886	90.22%	96	9.78%	0.332
	YES	15	83.33%	3	16.67%	
**NICU**	NO	683	90.22%	74	9.78%	0.816
	YES	218	89.71%	25	10.29%	
**NICU ventilation**	NO	778	90.15%	85	9.85%	0.893
	YES	123	89.78%	14	10.22%	
**Surfactant Given**	NO	755	90.31%	81	9.69%	0.614
	YES	146	89.02%	18	10.98%	
**Breastfed**	Exclusive	365	89.46%	43	10.54%	0.346
	Mixed	291	90.65%	30	9.35%	
	NO	304	91.02%	30	8.98%	
**Mother smoking during pregnancy**	NO	854	89.71%	98	10.29%	0.063
	YES	47	97.92%	1	2.08%	
**Patient on special milk/diet**	NO	868	89.86%	98	10.14%	0.167
	YES	33	97.06%	1	2.94%	
**Overcrowding**	NO	757	90.44%	80	9.56%	0.342
	YES	144	88.34%	19	11.66%	
**Parents smoking cigarettes or Shisha**	NO	254	91.70%	23	8.30%	0.295
	YES	647	89.49%	76	10.51%	
**Smoking inside home by parents or other households**	NO	697	90.28%	75	9.72%	0.719
	YES	204	89.47%	24	10.53%	
**Patient regular medications**	NO	735	90.41%	78	9.59%	0.499
	YES	166	88.77%	21	11.23%	
**Highest completed education of mother**	Diploma	73	82.02%	16	17.98%	**0.024**
	Primary school	102	93.58%	7	6.42%	
	Secondary school	443	90.22%	48	9.78%	
	University BSc	261	91.90%	23	8.10%	
	University Postgraduate	22	81.48%	5	18.52%	
**Highest completed education of Father**	Diploma school	71	88.75%	9	11.25%	0.681
	Primary school	125	91.91%	11	8.09%	
	Secondary school	447	90.30%	48	9.70%	
	University BSc	213	90.25%	23	9.75%	

	University Postgraduate	45	84.91%	8	15.09%	
**Chronic conditions**						
**Asthma**	NO	854	90.08%	94	9.92%	0.944
	YES	47	90.38%	5	9.62%	
**Bronchopulmonary dysplasia**	NO	900	90.09%	99	9.91%	0.740
	YES	1	100.00%	0	0.00%	
**Congenital heart disease**	NO	863	90.08%	95	9.92%	0.934
	YES	38	90.48%	4	9.52%	
**Neuromuscular disease**	NO	888	90.06%	98	9.94%	0.728
	YES	13	92.86%	1	7.14%	
**Other comorbidities ****	NO	801	90.20%	87	9.80%	0.759
	YES	100	89.29%	12	10.71%	
**Patient attending kindergarten or day care**	NO	824	90.65%	85	9.35%	0.066
	YES	77	84.62%	14	15.38%	
**Parent with history of atopic eczema**	NO	827	89.99%	92	10.01%	0.693
	YES	74	91.36%	7	8.64%	
**Siblings attending kindergarten or day care**	NO	725	90.29%	78	9.71%	0.690
	YES	176	89.34%	21	10.66%	
**Parent with history of asthma**	NO	823	89.85%	93	10.15%	0.377
	YES	78	92.86%	6	7.14%	
**Siblings with history of asthma**	NO	824	89.76%	94	10.24%	
	YES	77	93.90%	5	6.10%	
**Siblings <5 years living in the same household**	NO	366	89.71%	42	10.29%	0.729
	YES	535	90.37%	57	9.63%	

*Chi-square test, statistically significant at P < 0.05.
** other comorbidities include Global Developmental Delay (GDD), Reactive Airway Disease (RAD) present since birth, cerebral atrophy, hypothyroidism, Down Syndrome, and Chronic Lung Disease (CLD) with concurrent pulmonary hypertension, a large Ventricular Septal Defect (VSD), and heart failure. Additionally, hypotonia, with concerns raised about suspected congenital muscular dystrophy, and kidney disease.
Two sample t-test, statistically significant at <0.05.

Supplementary Table s3. Descriptive statistics of clinical findings by influenza positivity.

		Influenza				
		NO		YES		
		Count	Within Influenza result %	Count	Within Influenza result %	p-value*
**Chest X ray infiltrate**		579	64.3%	68	68.7%	0.382
**White blood cell count ($x10^9$/L)**	<4.0	10	1.1%	3	3.0%	**<0.001**
	4 to 10	295	32.7%	62	62.6%	
	>10.0	594	65.9%	34	34.3%	
**Cardiovascular**		9	1.0%	1	1.0%	0.992
**Low activity level**		265	29.4%	33	33.3%	0.418
**Apnea>10sec**		9	1.0%	1	1.0%	0.992
**Dehydration**		205	22.8%	28	28.3%	0.217
**Hypoxia (SaO_2 <92%)**		216	24.0%	18	18.2%	0.196
**Subcostal/intercostal retractions**		396	44.0%	35	35.4%	0.101

**Acute respiratory distress**	258	28.6%	24	24.2%	0.357
**Tachypnea**	481	53.4%	33	33.3%	**<0.001**
**Cyanosis**	73	8.1%	5	5.1%	0.283
**Pneumothorax/Atelectasis**	2	0.2%	1	1.0%	0.174
**Nasal Flaring**	17	1.9%	1	1.0%	0.533
**Overall oxygen need- Nasal cannula**	233	25.9%	23	23.2%	0.570
**Overall oxygen need- High flow**	29	3.2%	1	1.0%	0.221
**Overall oxygen need- Non-invasive ventilation**	58	6.4%	1	1.0%	**0.030**
**Overall oxygen need- Invasive ventilation**	8	0.9%	1	1.0%	0.903

***Chi-square, statistically significant at P < 0.05.**
